# Detection of MOG-IgG in Clinical Samples by Live Cell-Based Assays: Performance of Immunofluorescence Microscopy and Flow Cytometry

**DOI:** 10.3389/fimmu.2021.642272

**Published:** 2021-05-07

**Authors:** Amanda Marchionatti, Gisele Hansel, Gabriela Urbanski Avila, Douglas Kazutoshi Sato

**Affiliations:** ^1^ Neuroinflammation and Neuroimmunology Lab, Brain Institute of Rio Grande do Sul, Pontifical Catholic University of Rio Grande do Sul (PUCRS), Porto Alegre, Brazil; ^2^ School of Medicine, Graduate Program in Pediatrics and Child Health, Pontifical Catholic University of Rio Grande do Sul (PUCRS), Porto Alegre, Brazil

**Keywords:** myelin oligodendrocyte glycoprotein, cell-based assay, immunoglobulin-G, demyelinating disease, central nervous system

## Abstract

Human antibodies against Myelin Oligodendrocyte Glycoprotein (MOG) from immunoglobulin-G subclasses (MOG-IgG) have been recently associated with a new subgroup of neurological autoimmune diseases with distinct clinical characteristics from multiple sclerosis and neuromyelitis optica spectrum disorders. The use of MOG-IgG as a biomarker is an essential tool to assist in the diagnosis and clinical prognosis. The cell-based assay (CBA) is a methodology that expresses high levels of natively folded human MOG protein in the cell membrane being the methodology most used for clinical MOG-IgG diagnosis. However, there is still no consensus about the best approach to perform CBA to improve the results. The CBA using flow cytometry (CBA-FC) is an automated technique with objective quantification, reducing the subject of human bias that occurred at CBA using immunofluorescence (CBA-IF). In this study, we compared the performance of CBA-IF and CBA-FC as an acquisition tool analysis. The sera of 104 patients diagnosed with inflammatory Central Nervous System diseases were tested in both CBA-IF and CBA-FC. We used the dilution of 1:128 for CBA-IF and three different dilutions (1:20, 1:100, and 1:640) for CBA-FC. The CBA-FC and CBA-IF results had 88.5% agreement between assays and the CBA-IF titers by endpoint-dilution correlated with the CBA-FC titers. The highest serum dilution resulted in an increased CBA-FC specificity, but there was a reduction in the CBA-FC sensitivity. Our study showed that CBA-FC can be used in clinical practice as a diagnostic technique for MOG-IgG. In addition, in some specific cases, the combination of both techniques could be used as a tool to discriminate unspecific binding and overcome single assay limitations.

## Introduction

Myelin Oligodendrocyte Glycoprotein (MOG) is a protein exclusively expressed in the Central Nervous System (CNS). It is present in the outer membrane of the myelin sheath and oligodendrocytes, being easily accessible to the immune-mediated response. The MOG has been used for decades as an antigen to produce experimental autoimmune encephalomyelitis (1, 2). The human antibodies against MOG from immunoglobulin-G subclasses (MOG-IgG) have been recently associated with a new subgroup of neurological autoimmune diseases, with distinct clinical characteristics and prognosis from multiple sclerosis (MS) and aquaporin-4-IgG positive neuromyelitis optica spectrum disorders (NMOSD). Our group proposed the acronym MONEM to facilitate the identification of suspected cases as most the MOG-IgG^+^ patients present with attacks of Optic Neuritis (ON), Encephalitis, and/or Myelitis (3), but some other groups have used the term MOG-associated disorder (MOGAD) (4). The ON is the most common clinical manifestation followed by transverse myelitis (TM) (5–7). The presence of MOG-IgG also occurs in some patients that can be clinically diagnosed with aquaporin-4-IgG seronegative NMOSD, acute disseminated encephalomyelitis (ADEM), and cortical encephalitis (8–10). The MONEM cases are found in Caucasian and non-Caucasian paediatric and adult patients with a slight female predominance (6, 11, 12).

The MOG-IgG were initially detected by enzyme-linked immunosorbent assay (ELISA) or Western Blot, which were later discarded for clinical practice mainly due to low specificity. These methods usually use unfold and/or denatured protein unable to distinguish specific antibodies against conformational sensitive MOG antibodies. These methodology analyses led to false-positive results in MS patients and also healthy individuals. Therefore, the development of techniques that discriminate MOG-IgG binding to conformational and non-conformational epitopes was essential for use in clinical practice (3, 13, 14). The cell-based assay (CBA) uses cell lines transfected with plasmids encoding the human MOG sequence. This methodology expresses high levels of natively folded MOG protein in the cell membrane. The detection of MOG-IgG by CBA has been recently used for clinical diagnoses (15–17). Commercial kits with pre-fixed cells can be used, however, there may have some loss of sensitivity and specificity compared to the cell-based assays with live transfected cells (15). Despite recent efforts to find the best methodology for detecting MOG-IgG in clinical practice, there is no standardization among MOG-IgG CBA protocols from different laboratories.

In terms of detection methods for MOG-IgG, the CBA can be performed by indirect immunofluorescence microscopy (CBA-IF) or flow cytometry (CBA-FC) (18). Currently, the CBA-IF is widely used for the detection of autoantibodies, but it is a semi-quantitative technique and subject to human bias, even if performed by experienced professionals. On the other hand, CBA-FC is an automated technique with objective quantification, reducing human error. Nevertheless, CBA-FC requires strict standardization of the flow-cytometry parameters, with variation between devices and days. In addition, the fluorescence signals may vary according to the MOG expression and secondary antibody binding in each assay batch (19). Concerning the analysis methods, the MOG-IgG by CBA-FC is usually evaluated by the ratio or the delta of mean fluorescence intensity (MFI) comparing transfected and untransfected cells (20).

Some MOG-IgG assays from distinct centers were compared in a recent study (15), as well a direct comparison of MOG-IgG assays detected by CBA-IF and CBA-FC developed in the same research laboratory (21). However, there is still no consensus on what is the most reliable way to detect MOG-IgG, or what is the best dilution of serum for use in clinical diagnosis. Therefore, in the present study, we compared the performance of CBA-IF and CBA-FC analysis, using the serum of patients diagnosed with inflammatory CNS diseases, suspecting positivity of MOG-IgG.

## Materials and Methods

### Patients

Sera from 104 patients diagnosed with CNS inflammatory diseases, suspecting MOG-IgG^+^ were included in the study. The blood samples and the clinical data were collected in the Neuroinflammation and Neuroimmunology Lab between 2015 and 2019. All samples were analyzed by a blind researcher who had no access to clinical information. The present study was approved by the Ethics Committee from the Pontifical Catholic University of Rio Grande do Sul (CAAE 03402818.4.0000.5336).

### Transfection of Human Embryonic Kidney 293 (HEK293) cells

For live CBA, HEK293 cells (ATCC, LGC Standards GmbH, Wesel, Germany) were maintained in Dulbecco’s Modified Eagle’s Medium (DMEM) (Gibco, Life Technologies, NY, USA, Cat#12100-046) supplemented with 10% of Fetal Bovine Serum (FBS) (Gibco, Life Technologies, NY, USA, Cat#12657-029), 1% (100 U/mL) of penicillin/streptomycin (Gibco, Life Technologies, NY, USA, Cat#15140-122) and 0.1% (100 μg/mL) of gentamicin (Gibco, Life Technologies, NY, USA, Cat#15710-064) in the incubator (5% CO2; 37°C) until achieving 60% of confluence. Thereafter, the HEK293 cells were transfected with a plasmid containing full-length human MOG (FL-MOG) α1 isoform, using Fugene HD transfection reagent (Promega Corporation, WI, USA, Cat#E2311) according to manufacturer’s specifications. For live CBA-IF, HEK293 cells were transfected with a recombinant pIRES Ds-Red2 expression vector with full-length human MOG (FL-MOG). For live CBA-FC, HEK293 cells were transfected with a pEGFP-N1 plasmid containing FL-MOG fused to EGFP (MOG-EGFP). After 24 hours, the cells were trypsinized using 0.05% Trypsin-EDTA (Gibco, Life Technologies, NY, USA, Cat#15400-054), centrifuged, and resuspended in DMEM with geneticin G418 (Sigma-Aldrich, MO, USA, Cat#10131035) for selection, and maintenance of stably transfected cells.

### Immunofluorescence Microscopy

For live CBA-IF, MOG-DsRed transfected cells were trypsinized, centrifuged, resuspended in DMEM, and transferred to glass slides, and maintained overnight in the incubator. Then, the cells were washed once with phosphate buffer saline (PBS) pH 7.4 and incubated with 20 µl of serum (dilution 1:128) for 30 min at room temperature. The cells were washed twice with PBS and added 15 µl of anti-Human IgG Fc-specific Cross-Adsorbed, DyLight 488 (Thermo Scientific, Life Technologies, MA, USA, Cat#SA5-10134) at 1:500 dilution for 30 min. After, cells were washed 3 times, fixed, and added mounting medium with DAPI (Fluoroshield™ with DAPI, Sigma-Aldrich, MO, USA, Cat#F6057). The slide was covered and analyzed on a confocal microscope. The cut-off value for seropositivity was 1:128 as previously described (10). For positive samples, MOG-IgG titers were determined using serial two-fold endpoint dilutions. The endpoint titer was defined as the highest dilution that gives a positive fluorescence signal.

### Flow Cytometry

For each sample, 1 × 10^5^ MOG-EGFP transfected cells were harvested, washed twice with PBS pH 7.4, and incubated for 30 minutes at 4°C with patient serum. For the CBA-FC experiment, we used three different serum dilutions (1:20, 1:100, and 1:640). Thereafter, the cells were washed twice and incubated with Anti-Human IgG Fc-specific Cross-Adsorbed, DyLight 650 (Thermo Scientific, Life Technologies, MA, USA, Cat#SA5-10137) at 1:250 for 30 minutes at 4°C. The cells were washed three times, resuspended in PBS, and analyzed by flow cytometry (Attune NxT Flow Cytometer, Thermo Scientific, Life Technologies, MA, USA). Each sample analysis was performed in duplicate. For analysis, the optimal data acquisition gate was established and the binding was expressed as MFI. The MOG-IgG titers were analyzed by two methods: the ratio of MFI (rMFI), and the delta of MFI (ΔMFI) between MOG-expressing cells versus non-transfected cells. To establish the cut-off value, a threshold obtained from a cohort of negative MOG-IgG patients was used, determined by the mean of MFI plus four standard deviations of all the negative samples, both rMFI, and ΔMFI

### Statistical Analysis

Statistical analyses were performed using SPSS statistics 22.0 (IBM Corp. Armonk, NY, USA) and GraphPad Prism 5 (GraphPad Software, La Jolla, CA, USA). Flow cytometry data were analyzed using FlowJo™ Software 10 (Becton, Dickinson and Company, USA). We used Cohen’s kappa to evaluate the concordance between CBA-IF and CBA-FC analyses and Spearman’s nonparametric correlation for antibody titers by CBA-IF and CBA-FC. Receiver-Operating Characteristic (ROC) curve analysis was used to determine the performance of CBA-FC seropositivity using CBA-IF as a reference assay. A *p*-value < 0.05 was considered statistically significant.

## Results

### Patients: Demographic and Clinical Data

Amongst the 104 patients with samples, 94.2% of them had available clinical data. Based on the CBA-IF results, the median age at disease onset was 20 years (± 15.4 years), and 42.2% were female in the MOG-IgG^+^ group. In the MOG-IgG^-^ group, the median age at disease onset was 30 years (± 15.3 years), and 69.5% was female. About disease presentation, in the MOG-IgG^+^ group the majority of patients (65.9%) presented with ON, 14.7% had myelitis, 2.4% had both ON and myelitis, 7.3% were clinically diagnosed with seronegative NMOSD, 7.3% had ADEM, and 2.4% had epileptic seizures. The MOG-IgG^-^ group had a clinical diagnosis as follows: 25.9% had ON, 27.6% had myelitis, 3.5% were clinically diagnosed with seronegative NMOSD, 5.2% were diagnosed with AQP4-NMOSD. In the MOG-IgG^-^ group, 10.2% were aquaporin-4-IgG positive, with no aquaporin-4-IgG positive in the MOG-IgG^+^ group (for complete information see **Supplementary Material Table 1**).

### Analysis of Seropositivity by CBA-IF

**Figure 1:**
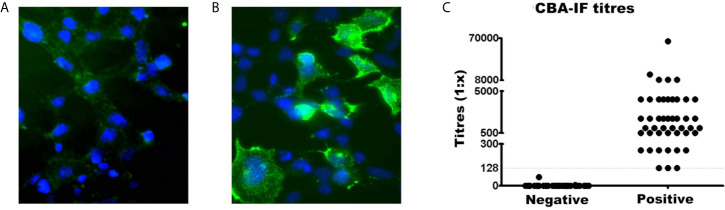
Comparison of MOG-IgG negative and positive samples by live cell-based assay immunofluorescence (CBA-IF). Representative fluorescence image of **(A)** negative and **(B)** positive samples. The green fluorescence DyLight 488 is an indicator of bound human serum MOG antibody. The blue fluorescence is a nuclear DAPI marker. Images were obtained using a confocal microscope with a 63× objective lens. **(C)** Represents the quantification of CBA-IF binding scores for negative and positive samples. The cut-off value for seropositivity (1:128 dilution) was represented by the dotted line. The titers of positive samples range from 1:128 to 1:65,556.

The serostatus of 104 patients tested using CBA-IF was 56.7% (n=59) seronegative (**Figure 1A**) and 43.3% (n=45) seropositive (**Figure 1B** **)**. The total IgG was evaluated and there was no difference between MOG-IgG^+^ and MOG-IgG^-^ samples (**Supplementary Material Table 2**). Two negative samples had a weak fluorescence emission below the cut-off (1:8 and 1:64). The titers of the MOG-IgG^+^ group range from 1:128 to 1:65,556 (**Figure 1C**).

### Analysis of Seropositivity by CBA-FC

For the CBA-FC evaluation, we used three serum dilutions (1:20, 1:100, and 1:640) analyzed under two distinct conditions, both calculations based on mean fluorescent intensity (ΔMFI = MFI positive - negative cells, and rMFI = MFI positive/negative cells). The CBA-FC groups were named 1 to 6, according to respective acronyms: CBA-FC1 (1:20, using ΔMFI analysis); CBA-FC2 (1:20, using rMFI analysis); CBA-FC3 (1:100, using ΔMFI analysis); CBA-FC4 (1:100, using rMFI analysis); CBA-FC5 (1:640, using ΔMFI analysis); CBA-FC6 (1:640, using rMFI analysis).

**Figure 2:**
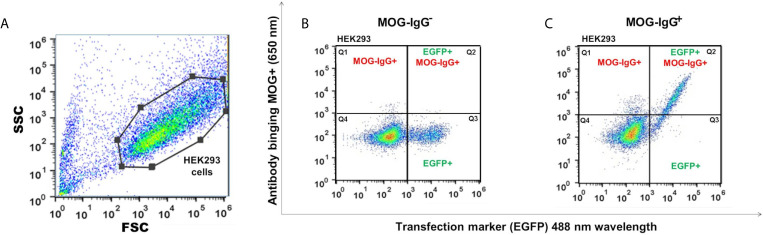
Scatter plots graphs of live cell-based assay flow cytometry (CBA-FC) analysis. **(A)** Representative flow cytometry strategy gating for HEK293 cells. The gate of flow cytometry data was based on forward scatter (FSC) versus side scatter (SSC), using a FlowJo™. Representative flow plots of **(B)** MOG-IgG^-^, and **(C)** MOG-IgG^+^ samples. The X-axis represents the MOG-EGFP transfection marker (488 nm) and the Y-axis represents the secondary anti-human Fc-IgG DyLight 650 nm antibody binding.

The MOG-IgG^+^ group represented 45.2%, 46.2%, 45.2%, 46.2%, 37.5%, and 39.4% in the CBA-FC 1 to 6, respectively (see **Figure 2** for an example of sample gate (2A), a seronegative (2B) and seropositive (2C) MOG-IgG by CBA-FC). In **Table 1**, we summarize the six CBA-FC analysis.

**Table 1:** Data of CBA-FC analysis.

	Analyses	Cutoff [*X*+(4× SD)]	MOG-IgG^+^	MOG-IgG^-^	MFI (range)positive samples
CBA-FC1	Dilution 1:20	377.40 [57.80 + (4 × 79.90)]	45.2% (n=47)	54.8% (n=57)	7353.97 (32469)
	ΔMFI				
CBA-FC2	Dilution 1:20	1.97 [1.17 + (4 × 0.20)]	46.2% (n=48)	53.8% (n=56)	17.58 (61)
	rMFI				
CBA-FC3	Dilution 1:100	268.90 [35.90 + (4 × 58.25)]	45.2% (n=47)	54.8% (n=57)	4928.67 (14148)
	ΔMFI				
CBA-FC4	Dilution 1:100	2.07 [1.11 + (4 × 0.24)]	46.2% (n=48)	53.8% (n=56)	18.76 (74)
	rMFI				
CBA-FC5	Dilution 1:640	50.28 [−7.48 + (4 × 14.44)]	37.5% (n=39)	62.5% (n=65)	694.63 (2905)
	ΔMFI				
CBA-FC6	Dilution 1:640	1.78 [0.90 + (4 × 0.22)]	39.4% (n=41)	60.6% (n=63)	6.02 (33)
	rMFI				

X, average; SD, standard deviation; MFI, mean fluorescent intensity, ΔMFI, delta MFI; rMF, ratio MFI.

### Comparison Between CBA-FC and CBA-IF

Comparing CBA-IF to CBA-FC, in all six analyses, 38 samples were seropositive and 54 were seronegative in both methods, corresponding to 88.5% of agreement. Only 12 patients (11.5%) had positive results only in one assay (CBA-FC or CBA-IF) and the data are summarized in **Table 2**. If the results were stratified into three subgroups, MOG-IgG^-^, MOG-IgG^+^ low-titer (1: 128 to 1: 512), and MOG-IgG^+^ high-titer (above 1:1024) based on CBA-IF, the agreement were 93.7%, 85.3%, and 95.7% respectively. Individually, the CBA-FC 5 and 6 had a better agreement in MOG-IgG negative samples (98.3%), CBA-FC 2 and 4 in the MOG-IgG positive samples with high-titer (100%), and CBA-FC1-4 in the MOG-IgG positive samples with low-titers (88.2%). It is very clear the reduction in agreement in MOG-IgG^+^ with low titer due to the proximity as the cut-off. The agreement data stratified into subgroups are summarized in **Table 3**.

**Table 2:** Inconsistent data between CBA-IF and CBA-FC.

Inconsistent samples	CBA-IF (Titers 1:x)	CBA-FC1 (ΔMFI)	CBA-FC2 (rMFI)	CBA-FC3 (ΔMFI)	CBA-FC4 (rMFI)	CBA-FC5 (ΔMFI)	CBA-FC6 (rMFI)
1	Negative	Positive (2053)	Positive (4.04)	Positive (3851)	Positive (6.28)	Negative (52)	Negative (1.69)
2	Negative	Positive (1395)	Positive (7.16)	Positive (1341)	Positive (2.27)	Negative (1)	Negative (1.5)
3	Negative	Positive (398.2)	Positive (4.99)	Positive (414.3)	Positive (3.17)	Negative (−9)	Negative (1.43)
4	Negative	Positive (1590)	Positive (5.47)	Positive (685.5)	Positive (5.02)	Negative (−30)	Negative (1.33)
5	Negative (1:64)	Positive (7258)	Positive (6.6)	Positive (2757)	Positive (4.48)	Positive (189)	Positive (2.03)
6	Positive (1:128)	Negative (84.5)	Negative (1.1)	Negative (123)	Negative (2.05)	Negative (−10)	Negative (1.43)
7	Positive (1:256)	Negative (267)	Negative (1.57)	Negative (127.5)	Negative (1.42)	Negative (−22)	Negative (1.34)
8	Positive (1:256)	Positive (3776)	Positive (7.48)	Positive (11005)	Positive (11.3)	Negative (48)	Positive (1.85)
9	Positive (1:256)	Positive (2660)	Positive (7.96)	Positive (1495)	Positive (5.46)	Negative (41)	Negative (1.73)
10	Positive (1:2048)	Negative (258)	Positive (2.51)	Negative (124)	Positive (2.1)	Negative (−1)	Negative (1.5)
11	Positive (1:2048)	Positive (10269)	Positive (9.24)	Positive (557.5)	Positive (2.09)	Negative (-184)	Negative (1.4)
12	Positive (1:2048)	Positive (3578)	Positive (24.6)	Positive (6421)	Positive (35.3)	Negative (34)	Positive (1.87)

**Table 3:** Concordance data stratified into subgroups.

	MOG-IgG^-^	MOG-IgG^+^	MOG-IgG^+^
		Low-titer (1:128 to 1:512)	High-titer (up to 1:1024)
CBA-FC1	91.5%	88.2%	96.4%
CBA-FC2	91.5%	88.2%	100.0%
CBA-FC3	91.5%	88.2%	96.4%
CBA-FC4	91.5%	88.2%	100.0%
CBA-FC5	98.3%	76.5%	89.3%
CBA-FC6	98.3%	82.3%	92.9%
**TOTAL**	**93.7%**	**85.3%**	**95.7%**

**Figure 3:**
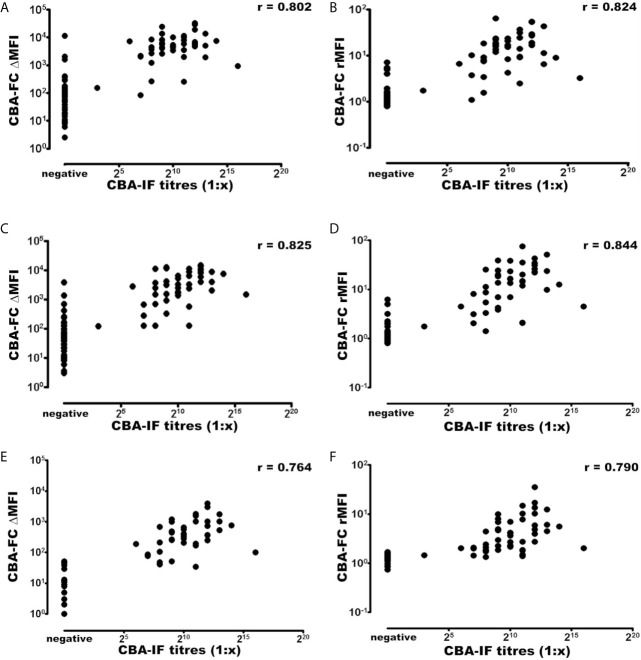
Correlation of MOG-IgG titers and MFI values determined by CBA-IF and CBA-FC assays. The analysis were generated using CBA-IF and **(A)** CBA-FC1, **(B)** CBA-FC2, **(C)** CBA-FC3, **(D)** CBA-FC4, **(E)** CBA-FC5, and **(F)** CBA-FC6. The graphs are expressed as logarithmic scale, and the correlation values were calculated using the non-parametric Spearman correlation and correlation coefficient (r) are shown in the graph.

Using the established CBA-IF as the reference standard the area under the curve (AUC) of the ROC curve was 0.959, 0.973, 0.968, 0.984, 0.931, and 0.946 in analyses CBA-FC 1 to 6 respectively (**Table 4**).The sensitivity analysis for CBA-FC1 to 6 were 93.3%, 95.6%, 93.3%, 95.6%, 84.4%, and 88.9%. The specificity was 91.5%, 91.5%, 91.5%, 91.5%, 98.3%, and 98.3% respectively. This data showed the high dilution (1:640) has greater specificity, but a slightly lower sensibility. The positive predictive value was 0.894, 0.896, 0.894, 0.896, 0.974, and 0.976 in analyses CBA-FC 1 to 6 respectively and the negative predictive value was 0.947, 0.964, 0.947, 0.964, 0.892, and 0.921. The kappa coefficient values used to assess the inter-method reliability were all above 0.8, indicating almost perfect agreement between methods (*p*<0.0001 for all analysis). In addition, the analysis of correlation comparing CBA-IF antibody titers to ΔMFI or rMFI used in CBA-FC analysis were 0.802, 0.824, 0.825, 0.844, 0.764 and 0.790 for CBA-FC1 to 6 respectively (Spearman’s rho; *p*<0.0001; **Figure 3**). The data demonstrate a strong positive correlation between the titers obtained in immunofluorescence microscopy and the analysis performed by cytometry.

**Table 4:** Data analysis comparing CBA-IF and CBA-FC.

	CBA-FC1	95% CI	CBA-FC2	95% CI	CBA-FC3	95% CI	CBA-FC4	95% CI	CBA-FC5	95% CI	CBA-FC6	95% CI
(AUC) of the ROC	0.959	0.918 to 0.999	0.973	0.943 to 1.000	0.968	0.939 to 0.996	0.984	0.967 to 1.000	0.931	0.870 to 0.993	0.946	0.896 to 0.997
Sensitivity	0.933	0.807 to 0.983	0.956	0.836 to 0.992	0.933	0.807 to 0.983	0.956	0.836 to 0.992	0.844	0.699 to 0.930	0.889	0.752 to 0.958
Specificity	0.915	0.806 to 0.968	0.915	0.805 to 0.968	0.915	0.806 to 0.968	0.915	0.806 to 0.968	0.983	0.897 to 0.999	0.983	0.897 to 0.999
Positive Predictive Value	0.894	0.761 to 0.960	0.896	0.766 to 0.961	0.894	0.761 to 0.960	0.896	0.766 to 0.961	0.974	0.849 to 0.999	0.976	0.856 to 0.999
Negative Predictive Value	0.947	0.845 to 0.986	0.964	0.866 to 0.994	0.947	0.845 to 0.986	0.964	0.866 to 0.994	0.892	0.785 to 0.952	0.921	0.817 to 0.970
Kappa coefficient values	0.844	0.740 to 0.948	0.864	0.767 to 0.961	0.844	0.740 to 0.948	0.864	0.767 to 0.961	0.841	0.736 to 0.946	0.881	0.789 to 0.973
Spearman’s coefficient values	0.802	0.718 to 0.864	0.824	0.748 to 0.879	0.825	0.749 to 0.880	0.844	0.776 to 0.893	0.764	0.666 to 0.836	0.790	0.700 to 0.855

Comparing ΔMFI and rMFI analysis, the rMFI had better results with 10 inconsistent results contrasting with 12 inconsistent samples by ΔMFI, representing 90.4% of agreement. Looking at the inconsistent samples, the five negative samples in immunofluorescence, only one had positive data at CBA-FC6 (high dilution). This specific sample had a title of 1:64 at immunofluorescence microscopy analysis, a value below the cut-off. In the inconsistent positive samples analyzed CBA-IF, all of them were negative in CBA-FC5. These data indicate that CBA-FC5 has a high specificity, but lower sensitivity. Evaluating CBA-IF and CBA-FC4, the agreement increased to 93.2%, with the highest AUC value and Spearman’s coefficient. Five samples were positive by CBA-FC4 and negative by CBA-IF. On the other hand, only two samples were negative using CBA-FC4 analysis comparing to positive results by CBA-IF, both with low-titers (1:256). The CBA-FC4 data showed the highest specificity (91.5%) and very high sensibility (95.6%) among the CBA-FC analysis.

## Discussion

The MOGAD or MONEM is a new subgroup of neurological autoimmune disorders, distinct from MS and aquaporin-4-IgG^+^ NMOSD. The MOG-IgG has been increasingly used as a biomarker in inflammatory CNS disorders, helping to improve diagnostic accuracy and stratify prognosis, as well as promoting the development of new treatments targeting specific molecules (3, 17).

In this study, we examined the determination of MOG-IgG using live CBA performed by indirect immunofluorescence microscopy and multiple flow cytometry conditions. Using the CBA-IF as the standard, we compare the CBA-FC methodology using ΔMFI and rMFI in three different dilutions (1:20, 1:100, and 1:640). Our results indicate a high agreement between CBA-IF and CBA-FC with all kappa coefficient values close to 0.8. There were undoubtful MOG-IgG positive with high-titers and negative patients in all assays, with an agreement of 88.5% of the total of samples. However, twelve samples had discrepancies in the results from the CBA-IF and CBA-FC.

Further analyzing inconsistent samples, five of them were seronegative by CBA-IF while seropositive by CBA-FC1 to 4. The flow cytometry graphs have shown a distinct MOG-EGFP profile compared to strongly positive samples (data not shown). One of these patients was AQP4-IgG^+^ NMOSD with high background and less likely to be a case of double antigen positivity. There are very rare cases of double positivity, and most of these cases have a very high titer from one antibody and the other near the cut-off values (22). Evaluating the sample in question, we believe that it is not a case of double antigen positivity, but an unspecified fluorescence staining. Four positive samples that occurred in low-dilutions (1:20 and 1:100) were negative when tested in higher dilution (1:640). The presence of several antigens and proteins in the serum may contribute to nonspecific binding. Besides, the amount of sample added in the assay may contribute to this nonspecific effect, evidenced in the analysis by flow cytometry with smaller dilutions. In a previous study, concerns about the use of low serum dilutions were detected due to false seropositivity in healthy patients (14). Thus, specificity can be significantly increased by using higher dilutions. Studies have shown that intermediate dilutions, such as 1:200 (23, 24), 1:320 (20), could increase specificity without reducing sensitivity. Two patients were seropositive at CBA-IF and seronegative at CBA-FC, but these patients had MOG-IgG titers close to the cut-off. High dilutions (up to 1:640) in CBA-IF were used as cut-off values for seropositivity (21), so it might be possible that unspecific binding and increased slide background leading to reading errors in the CBA-IF that did not happen in the CBA-FC. Four other samples were seropositive at CBA-IF and CBA-FC1-4, but negative by CBA-FC 5 and 6 analysis, demonstrating that the sensitivity can be reduced using higher dilutions. Another sample was seropositive in the CBA-IF and CBA-FC 2 and 4 using rMFI but negative in other analysis. In this case, the high background in non-transfected cells reduced the ΔMFI below the cut-off limits, as the difference in the anti-human IgG secondary antibody binding between transfected cells and non-transfected cells is very small when there is a high background (25). In our study, the rMFI analysis had a slightly higher agreement with CBA-IF than ΔMFI analysis. However, the study by Tea et al. (2020) revealed that the ratio analysis also has the sensitivity reduced by high background binding (26). Therefore, in a few cases, we may need to perform combined analysis and review the flow-cytometry plots to increase the accuracy of the assay results.

All ROC curves from the six CBA-FC assays having the CBA-IF as a reference assay demonstrated high sensitivity and specificity. Amongst the six CBA-FC assays the CBA-FC 4 using a 1:100 dilution and rMFI resulted in the highest AUC index. The most sensitive CBA-FC were those evaluated by the ratio (CBA-FC 2 and 4), while the specificity was slightly higher in the analysis with the CBA-FC using higher serum dilutions (CBA-FC 5 and 6). This corroborates with our results that the rMFI analysis with higher dilutions results in high accuracy. In our study, CBA-FC provides a relatively good agreement with CBA-IF titers and CBA-FC values for all analyses, but other groups reported discrepant results (25, 27). Compared with CBA-IF, the CBA-FC4 yielded the highest accuracy among the CBA-FC analysis. In addition, the CBA-FC4 values ​​provide a good agreement with the CBA-IF titers, suggesting to be the closest CBA-FC assay from the well-established CBA-IF.

his study has some limitations: (1) The sample size is relatively small, as the disorder caused by MOG-IgG is rare; (2) The cut-off point was performed with previously determined negative patients and not in a control group with healthy individuals; (3) A single cut-off point for CBA-FC was defined for all samples, while some authors recommend adjusting cut-off in every new experiment.

Both CBA-IF and CBA-FC are used in clinical practice, but each of them has different advantages and limitations. The CBA-IF is more commonly used for the detection of MOG-IgG in clinical practice, as it requires fluorescent microscopy available in most laboratories. The CBA-FC requires a multichannel flow cytometer which allows the simultaneous analysis of every single cell for the expression of MOG and the fluorescent intensity for anti-human IgG secondary antibody bound in the cell membrane in distinct channels. The antibody titers by CBA-FC can be calculated in the same data by different methods as described in this study. Furthermore, several samples can be analyzed on the same day and the data can be saved and re-analyzed by other statistical methodologies in the future.

In conclusion, our study showed that CBA-FC can be used in clinical practice as a diagnostic technique for MOG-IgG. The CBA-FC4 using a serum dilution of 1:100 and rMFI had a higher concordance with live CBA-IF. In addition, in some specific cases, the combination of both techniques could be used as a tool to discriminate unspecific binding and overcome single assay limitations.

## Data Availability Statement

The raw data supporting the conclusions of this article will be made available by the authors, without undue reservation.

## Ethics Statement

The studies involving human participants were reviewed and approved by Ethics Committee of the Pontifical Catholic University of Rio Grande do Sul (CAAE 03402818.4.0000.5336). The patients/participants provided their written informed consent to participate in this study.

## Author Contributions

Conceptualization, AM and DKS Data curation, AM, GUA, and GH. Formal analysis, AM and GH. Funding acquisition, DKS. Investigation, AM, GUA, and GH. Methodology AM GH, and DKS. Project administration, AM, GH, and DKS. Resources, DKS. Supervision, DKS. Visualization, AM, GH, and DKS. Writing—original draft, AM. Writing—review and editing, GH and DKS. All authors contributed to the article and approved the submitted version.

## Funding

This work was funded by grants by Coordenação de Aperfeiçoamento de Pessoal de Nivel Superior (CAPES)/Brazil– Finance Code 001. A.M. was supported by a scholarship from CAPES/Brazil. G.H. is a postdoctoral fellow supported by the CAPES-PRINT. D.K.S. received a scholarship from the Ministry of Education, Culture, Sports, Science and Technology (MEXT) of Japan, a Grants-in-Aid for Scientific Research from the Japan Society for the Promotion of Science (KAKENHI 15K19472), research support from CNPq/Brazil (425331/2016-4 and 308636/2019-8), TEVA, Merck, Biogen and Euroimmun AG for investigator-initiated studies, and speaker honoraria from Biogen, Novartis, Genzyme, TEVA, Merck, Roche and Bayer, and participated in advisory boards for Biogen, Roche, TEVA, and Merck.

## Conflict of Interest

The authors declare that the research was conducted in the absence of any commercial or financial relationships that could be construed as a potential conflict of interest.

## Supplementary Material

Supplementary Table 1 Demographic and Clinical Data.

Supplementary Table 2 Total IgG analysis data.
