# Supplementary material for: Detection of MOG-IgG in Clinical Samples by Live Cell-Based Assays: Performance of Immunofluorescence Microscopy and Flow Cytometry
Source: Front Immunol. 2021 May 7;12:642272. doi: 10.3389/fimmu.2021.642272 (PMC8137838; doi:10.3389/fimmu.2021.642272)
Supplement: Supplementary Table 1 — Demographic and Clinical Data. [file Table_1.docx]

Supplementary Material

# Supplementary Tables

**Supplementary Table 1:**

|  | **Samples for MOG-IgG testing (N=104)** | |
| --- | --- | --- |
| CBA-IF Results, (N) | MOG-IgG+ (N=45) | MOG-IgG- (N=59) |
| Age at onset of symptoms (years), median | 20 | 30 |
| Blood collection age (years), median | 25 | 32 |
| Female, N (%) | 19 (42.2) | 41 (69.5) |
| Clinical diagnosis, % |  |  |
| - Optic neuritis | 65.9 | 25.9 |
| - Myelitis | 14.6 | 27.6 |
| - ON and myelitis | 2.4 | - |
| - Seronegative NMOSD | 7.3 | 3.5 |
| - AQP4-NMOSD | - | 5.2 |
| - CIS | - | 1.7 |
| - ADEM | 7.3 | 5.2 |
| - Multiple sclerosis | - | 15.5 |
| - Encephalitis / Seizures | 2.4 | 6.9 |
| - Rhombencephalitis | - | 1.7 |
| - Acute polyneuropathy | - | 1.7 |
| - HAM | - | 1.7 |
| - Brain swelling injury | - | 1.7 |
| - Peripheral facial paralysis | - | 1.7 |
|  |  |  |
| AQP4-IgG+, % | - | 10.2 |

Abbreviations: ON = optic neuritis; NMOSD = Neuromyelitis optica spectrum disorder; AQP4 =aquaporin-4; CIS = clinically isolated syndrome; ADEM = acute disseminated encephalomyelitis; HAM = HTLV-I associated myelopathy

**Supplementary Table 2:**

|  | Total IgG (mg/ml) | | |
| --- | --- | --- | --- |
|  | **Mean** | **Range** | **Std Deviation** |
| MOG-IgG^+^ | 34.0 | 18.8 | 4.1 |
| MOG-IgG^-^ | 32.1 | 15.3 | 3.6 |

The total IgG were measured in the NanoDrop^TM^ Lite (Thermo Scientific, Life Technologies, MA, EUA), according to the manufacturer's specifications.
